# Phospholipases D: making sense of redundancy and duplication

**DOI:** 10.1042/BSR20181883

**Published:** 2019-06-28

**Authors:** Andrew J. Morris

**Affiliations:** Division of Cardiovascular Medicine, University of Kentucky College of Medicine and Lexington Veterans Affairs, Lexington, KY, U.S.A.

**Keywords:** Drosophila melanogaster, phospholipases, phosphatidic acids

## Abstract

Why have two genes when one would suffice? Evolutionary pressure means that biology, unlike government, is generally intolerant of wasted effort. Therefore, when multiple genes exist presumably they are there to provide some benefit to the organism even if that benefit is not immediately obvious to us scientists. A recent report from Raghu and colleagues (*Biosci. Rep.* (2018) **38**, pii: BSR20181690) [1] sheds some light on one possible reason for the existence of two Phospholipases D genes in chordates when only one is present in invertebrates.

Phospholipase Ds (PLDs) are phosphodiesterases that hydrolyze the diester phosphate bond of glycerol phospholipids to produce phosphatidic acid and the corresponding lipid headgroup. In eukaryotes, the best characterized enzymes of this type are selective for phosphatidylcholine. Budding yeast contain a single PLD gene, which encodes an enzyme that has no discernible function during vegetative growth (but can become essential when certain other genes involved in lipid metabolism are inactive), but that is required for sporulation [2]. This function requires the catalytic activity of the enzyme, which is essential for directing vesicular transport to form the membrane surrounding the nucleus of the nascent spore. Mammalian genomes invariably contain two PLD genes termed PLD1 and PLD2 [3,4]. Although these enzymes catalyze the same biochemical reactions and have largely overlapping expression patterns they differ markedly in their subcellular localization and in the mechanisms by which their catalytic activities are regulated [5]. In the accompanying paper, Raghu and colleagues conducted a phylogenetic analysis of PLD homologs revealing that the presumptive gene duplication that gave rise to PLD1 and PLD2 happened during the evolution of chordates. Insects contain a single PLD gene that appears closer to chordate PLD1 than to PLD2. In *Drosophila*, this single PLD gene is required for photoreceptor function and phototransduction (light sensing) because this light-activated enzyme is required for integrity of the apical plasma membrane and for recycling of vesicles to the apical plasma membrane after light-induced endocytosis. These homozygous PLD-mutant *Drosophila* have no detectable PLD activity yet they develop normally and have no discernable phenotype save the phototransduction defect discussed above [6].

This finding is broadly consistent with reports about PLD deficiency in other organisms where normal development and physiological functions are generally unimpaired but important phenotypes are revealed in settings of stress and disease. For example, mice with deficiency of PLD1 exhibit defects in platelet activation while inactivation of either PLD1 or PLD2 impairs some aspects of macrophage activation and endocytosis [7]. Mice with genetic inactivation of both PLD1 and PLD2 are also viable but exhibit defects in brain development and cognitive function [8]. These relatively underwhelming effects of whole body PLD1 and PLD2 deficiency may reflect compensatory mechanisms [5]. The PLD lipid product, phosphatidic acid, is widely implicated as the mediator of PLD-dependent signaling and regulatory processes. Phosphatidic acid can be generated by *de novo* synthesis or the actions of diacylglycerol kinases. Indeed, while budding, yeast lacking the single PLD have no phenotype during vegetative growth, phenotypes are apparent when other genes involved in lipid synthesis and transport are also inactivated. Further evidence that compensatory mechanisms blunt or mask phenotypes associated with PLD1 and PLD2 deficiency also comes from studies using acute genetic (RNA interference or over expression of inactive mutants that may have dominant negative or interfering effects) or chemical biology approaches [9]. The latter of these methods relied heavily on the unique ability of PLD to catalyze transphosphatidylation reactions using primary alcohols instead of water in the hydrolysis step of their catalytic cycle. This reaction generates phosphatidylalcohols and both offers a way to monitor PLD activity and also an approach to attenuate PLD-dependent PA production. Inhibitory effects of primarily alcohols (but not secondary alcohols which are not PLD substrates) inhibit multiple cellular processes including vesicular transport, regulation of the actin cytoskeleton and cell growth. These processes may therefore be regulated by PLD activity. This relatively unsophisticated approach has been superseded by the availability of potent selective small molecule PLD inhibitors in some cases with selectivity for PLD1 and PLD2. To some extent, studies using these chemical tools have recapitulated and extended observations made using less sophisticated approaches.

These efforts generally support the idea that PLD inhibition could be a useful therapeutic approach in settings where PLD-dependent pathways are aberrantly up-regulated, for example cancer, thrombosis and neurodegeneration. The fact that genetic deficiency of PLD is so well tolerated at least in animal models is certainly encouraging. However, validation of PLD as a therapeutic target in these disease settings through studies of normal human genetic variation or the identification of individuals with rare PLD-inactivating mutations is still lacking. More fundamentally, the redundant and duplicate functions revealed in the above studies and the possibility that PLD deficiency can be compensated for by alternative pathways for generation of PA raises important unanswered questions about the significance of the presence of PLD1 and PLD2 genes in chordates

The PLD-deficient *Drosophila* described by Raghu and colleagues provide an appealing and unique model system to examine the functional differences between mammalian PLD1 and PLD2 by comparing their abilities to complement the PLD-dependent phototransduction defect. This was accomplished by transgenic overexpression of the mammalian enzymes using a reporter system that restricted expression to adult photoreceptor cells. When PLD1 was overexpressed in wild-type *Drosophila*, this recapitulated the light-dependent retinal degradation observed with overexpression of the single *Drosophila* PLD gene. However, this light-dependent retinal degeneration was not observed with overexpression of PLD2 suggesting that PLD1 and PLD2 have different functions in this process. These differences between PLD1 and PLD2 were further explored by evaluating their ability to complement genetic deficiency of *Drosophila* PLD. In these experiments, PLD1 was able to completely suppress the light-dependent retinal degeneration phenotype of *Drosophila* PLD mutants. Suppression of this phenotype was only partial and slower in onset in *Drosophila* PLD mutants overexpressing PLD2. In mammalian cells, PLD1 and PLD2 exhibit distinct localization patterns. While PLD1 exhibits marked localization to intracellular membrane compartments, particularly the Golgi apparatus PLD2 is localized predominantly to the plasma membrane. *Drosophila* PLD localizes to a region close to the plasma membrane at the base of microvilli. PLD1 exhibited a broadly overlapping expression pattern with *Drosophila* PLD, while PLD2 did not and was excluded from this region of the photoreceptors. Together these observations raise the possibility that different localization patterns contribute to or account for the differential abilities of PLD1 and PLD2 to rescue *Drosophila* PLD deficiency.

PLD-catalyzed hydrolysis of phospholipids generates two products: the lipid head group and phosphatidic acid. Although PLD enzymes with broader substrate selectivity are present in plants and some bacterial PLD1, PLD2 and *Drosophila* PLD are phosphatidylcholine-specific enzymes. The water-soluble choline product is not considered the primary PLD-generated signal. On the other hand, phosphatidic acids are implicated as signaling molecules with targets that include protein kinases and small GTPase regulators and may alter the properties of biological membranes to impact on membrane dynamics in settings that include vesicular transport or actin-dependent re-organization of the plasma membrane during cell motility [10].

Phosphatidic acids can exhibit considerable structural diversity, primarily related to the length, saturation and linkage of the radyl hydrocarbon chains. These are most commonly esterified fatty acids. These phosphatidic acid responsive target molecules exhibit some selectivity for different phosphatidic acid molecular species at least *in vitro*. Effects of phosphatidic acid on membrane structure, for example membrane curvature, also exhibit chain length specific effects. For example, shorter chain phosphatidic acids may promote membrane curvature.

PLD1 and PLD2 exhibit some moderate selectivity for different phosphatidylcholine substrate molecular species at least *in vitro* but despite the potential importance of this selectivity for PLD function, we know very little about the substrate molecular species selectivity of PLD *in vivo*. To examine this possibility Raghu and colleagues developed HPLC-coupled tandem mass spectrometry methods that could detect and quantify different phosphatidic acid molecular species. These methods rely on monitoring product ions generated by fragmentation of the corresponding phosphatidic acid anions that correspond to loss of one of the acyl chains.

Although this method cannot discriminate between sn1- and sn2-substituted fatty acids, it does allow deduction of the chain length and saturation of the individual fatty acid substituents in the parent phosphatidic acid molecule. Application of this approach revealed that PLD1 and PLD2 generated distinct patterns of phosphatidic acid molecular species and that the pattern of species restored by PLD1 expression in *Drosophila* PLD mutants was substantially more similar to that observed in wild-type photoreceptors than that observed with overexpression of PLD2. The mechanism responsible for these differences is not clear and may relate to the differential localization of PLD1 and PLD2 with distinct phosphatidylcholine substrate rather than intrinsic differences in molecular species substrate selectivity. However, this finding suggests that in addition to the differences in localization differences in molecular species of phosphatidic acid might account for or contribute to the differential functions of PLD1 and PLD2 in complementation of *Drosophila* PLD deficiency. More work is needed to examine these possibilities.

In summary, a presumptive gene duplication event that occurred early during the evolution of chordates generated two PLD genes that encode proteins with the same enzymatic activity but differences in localization, regulation and possibility substrate selectivity. While lower organisms, including *Drosophila* can function with a single PLD gene, PLD1 and PLD2 are clearly not functionally redundant as evidenced by loss of function studies that include gene knockouts and chemical inhibitors. This new report from Raghu and colleagues augments this work by suggesting differences in localization and substrate selectivity may contribute to these functional differences between PLD1 and PLD2. Because PLD1 and PLD2 deficiency is relatively well tolerated in mammals taken together, these findings further support the concept that targeting PLD1 and PLD2 or their downstream signaling targets may be an effective therapeutic approach in settings such as cardiovascular disease and cancer where PLD activity is elevated or aberrantly regulated.
